# Comprehensive Analysis Reveals PTK6 as a Prognostic Biomarker Involved in the Immunosuppressive Microenvironment in Breast Cancer

**DOI:** 10.1155/2022/5160705

**Published:** 2022-11-09

**Authors:** Lili Wang, Shuimei Luo, Ziming Wang, Yiqiang Huang, Yang Luo, Xianhe Xie

**Affiliations:** ^1^Department of Breast Medical Oncology, Clinical Oncology School of Fujian Medical University, Fujian Cancer Hospital, Fuzhou 350014, China; ^2^Department of Oncology, Molecular Oncology Research Institute, The First Affiliated Hospital of Fujian Medical University, Fuzhou 350005, China; ^3^Fujian Key Laboratory of Precision Medicine for Cancer, The First Affiliated Hospital of Fujian Medical University, Fuzhou 350005, China

## Abstract

The significant mortality rate that is currently experienced by female breast cancer (BC) patients highlights the importance of locating potent and dependable biomarkers in BC patients. Over the past few years, a number of studies have demonstrated that PTK6 was dysregulated in a variety of cancers. However, its expression and the clinical importance it may have in patients with BC have not been explored. Based on datasets from the TCGA database and GTEx database, we studied the expressions and functions of PTK6 across 33 different kinds of cancer. In this study, we investigated the differential expression of PTK6 in tumor tissue compared to nontumor tissue as well as in various stages of cancer. ROC assays were used to conduct an investigation into the diagnostic potential of PTK6 in BC. After that, the Kaplan-Meier method, univariate analysis, and multivariate analysis were carried out in order to investigate the PTK6 gene's potential prognostic significance in patients with BC. ssGSEA was utilized in order to conduct an investigation of the immune infiltration. In this study, we discovered that the expressions of PTK6 were significantly raised in the majority of different types of malignancies, including BC. The diagnostic value of PTK6 expression was validated by ROC tests, demonstrating an AUC greater than 0.7. A positive PR, ER, and HER2 status was found to be related with high expression levels of PTK6. According to the results of a survival analysis, patients who had a high level of PTK6 expression had a shorter overall survival time than those who had a low level of PTK6 expression. Besides, we observed that PTK6 expressions were positively correlated with the abundance of NK CD56bright cells and Th17 cells and negatively correlated with that of Th1 cells, macrophages, B cells, T cells, aDC, DC, cytotoxic cells, Tem, TFH, NK CD56dim cells, Treg, and Tgd. In conclusion, PTK6 expression was found to be linked with the clinical phenotype of BC, and as a result, this finding may have consequences for the diagnosis, prognosis, and treatment of individuals with BC.

## 1. Introduction

Breast cancer (BC) is the most common cancer type in women, accounting for approximately 25% of new cancer cases [1, 2]. Although there have been great advancements in the diagnosis and treatment of breast cancer, recent data have revealed that the incidence and mortality rates of the disease are significantly increasing, with an estimated 278,000 novel cases and 64,000 deaths in China [3, 4]. The estrogen receptor positive subtype of breast cancer is the most prevalent form of the illness and accounts for close to 75% of all cases [5]. Endocrine therapy, molecular targeted therapy, radiation therapy, chemotherapy and surgery, and other forms of treatment are some of the options available for patients with ER+ breast cancer [6]. Even though there have been significant advances in treatments and even systemic treatments, the 5-year overall survival rate for breast cancer is still quite low [7]. This is especially the case when the cancer has spread to other parts of the body. BC is a complex disease, and standard diagnosis and treatment do not always produce desirable results in some patients because of the disease's diversity [8, 9]. Hence, there is an immediate need for innovative molecular biomarkers that are more reliable and can be used for the diagnosis, therapy, and forecasting of the prognosis of patients who have BC.

Protein tyrosine kinase 6 (PTK6) is a nonreceptor tyrosine kinase composed of an amino-terminal SH3 domain, SH2 domain, and carboxyl-terminal kinase domain [10]. During the first phase of our investigation, a functional genomic screen aiming to locate regulators of anchorage-independent survival helped us zero in on PTK6 as a crucial mediator of anoikis resistance in breast cancer cells [11, 12]. Over the course of the past few years, a number of additional PTK6 substrates have been discovered. Sam68 was the first substrate that was identified as being phosphorylated by PTK6, and it was demonstrated that PTK6 had a detrimental effect on the RNA-binding activity of Sam68 [13]. There is a possibility that PTK6 will have an effect on the post-transcriptional control of gene expression. PTK6 is a nonreceptor tyrosine kinase that is found to be amplified or highly expressed in a wide variety of human malignancies, including lung, breast, ovarian, and prostate cancer [14–16]. In addition, several studies have reported the role that PTK6 played in a variety of cancers. For instance, Liu et al. reported that PTK6 was distinctly increased in colorectal cancer samples and performs a stimulatory role in the growth and drug resistance of colorectal cancer cells in cellular experiments [17]. This is the case regardless of whether the cells are cultured or present in the body. Interacting with JAK2 and phosphorylating it to promote the JAK2/STAT3 pathway is one way that PTK6, and particularly phosphorylated PTK6, might enhance the stemness of colorectal cancer cells. Xu and his group reported that when compared to the normal controls, the expression of PTK6 is much higher in bladder cancer tissues. PTK6 overexpression was strongly linked to the T classification, the N classification, the grade, the recurrence of bladder cancer, and a poor clinical outcome in patients with the disease. In addition, bladder cancer cells were significantly less able to proliferate and migrate after having their expression of knocked down [18]. According to the studies presented above, PTK6 might be an oncogene in some cancers. However, the function of PTK6 in BC remain largely unclear.

Immune checkpoint blockade drugs have recently brought about a revolutionary shift in the anticancer treatment that is currently available for a diverse spectrum of malignancies [19]. About twenty percent of the samples, according to the results of the preclinical tests, showed an objective response, which suggested that immune checkpoint inhibitors may give unique insight into the therapeutic intervention and decision-making process regarding BC [20, 21]. The immune cells can either prevent tumor growth or stimulate tumor growth, and they may play a significant role as key participants in the TME [22, 23]. It is important to develop immune indicators that could predict treatment success and prognosis since the immune contexture has a substantial influence on the outcome of immunity therapy [24, 25]. This situation is because of the peculiarities of the immune contexture. At this point in time, the grade and stage of the tumors are often used as criteria for determining the prognosis of samples. According to the findings of a number of studies, the tumor mutation burden (TMB), which refers to the number of somatic coding errors like base substitutions, insertions, or deletions that occur across one million bases, is considered to be a promising indicator for predicting the patient's level of responsiveness to immune checkpoint blockade (ICB) [26, 27]. Finding innovative methods to evaluate prognosis and clinical outcome is beneficial for both the evaluation of patients' chances of survival and the treatment of BC patients.

We used RNA-seq data from the TCGA datasets, along with statistical and bioinformatics methods, such as analysis of differentially expressed genes (DEG), Kaplan-Meier (KM) survival analysis, Cox and logistic regression analysis, and single-sample gene set enrichment analysis (ssGSEA), to better understand the roles that PTK6 played in the progression of BC.

## 2. Methods and Materials

### 2.1. Data Collection

The TCGA datasets have profiled and evaluated a massive collection of clinical and molecular data relating to over 10,000 cancer patients suffering from 33 distinct forms of cancer. Transcriptome RNA-seq data of 33 cancers were extracted from the TCGA database (https://portal.gdc.cancer.gov/). In this study, a total of 1222 female breast cancer patients were utilized, 1109 of which provided specimens of tumors and 113 of whom provided specimens of normal breast tissue. From that group, 930 patients who had finished all of their follow-up appointments were chosen for the subsequent analyses. The clinical characteristics of BC patients are shown in Table 1.

### 2.2. Identification of Differently Expressed Genes (DEGs) in BC

In order to conduct a differential expression analysis of 1109 BC samples and 113 nontumor samples, the “limma” package of R was utilized. Samples with an adjusted false discovery rate *p* < 0.05 and ∣log fold change (FC) | >2 were considered as the threshold points for DEGs.

### 2.3. Functional Enrichment Analysis

The “clusterProfiler” R package was applied to carry out Kyoto Encyclopedia of Genes and Genomes (KEGG) pathway and Gene Ontology (GO) analyses based on the DEGs (∣log2FC∣≥2, FDR < 0.05) between BC specimens and nontumor specimens [28]. The parameters of the clusterProfiler R software package R were as follows: ont = all, *p* value − cutoff = 0.05, and *q* value − cutoff = 0.05.

### 2.4. Analysis of PTK6 Expression in Cancers

The TCGA and Genotype Tissue Expression (GTEx) projects provided the information that was used to determine the differential expression of PTK6 in tumor specimens and normal specimens. A tissue bank and data resource known as GTEx (http://gtexportal.org) was established by the National Institutes of Health (NIH) Common Fund. Plotting was done using log2 (TPM+1) converted expression data, which was our choice for the parameter selection process.

### 2.5. Analysis of the Association of PTK6 with Clinicopathological Features

The link between mRNA expressions of PTK6 and clinicopathological parameters, such as the age of the patient, the patient's ER status, the patient's HER2 status, the patient's histological type, M stage, N stage, pathologic stage, PR status, and T stage, was also investigated. The Student's test was used as the comparing method in the aforementioned research, and a *p* value of less than 0.05 was considered to be statistically significant.

### 2.6. PTK6 Expression Analysis and Survival Analysis

The original gene expression data were sorted and combined using the computer language Perl, and the limma tool of the R software was used to extract the PTK6 expression data from the dataset. For the purpose of visualizing the retrieved data and producing scatter difference diagrams, the limma and beeswarm packages were put to use. The Perl programming language was utilized in order to extract survival statistics from clinical data and get rid of the data that had information that was missing regarding survival time and survival status. Following that, the information regarding complete survival was matched with the data regarding BRCA expression. The BRCA mRNA expression level was classified into two groups according to its position relative to the median expression value (high-PTK6 expression group and low-PTK6 expression group). For the purpose of visualization, the survival package of the R software was utilized, and the Kaplan-Meier survival curve was obtained. In order to evaluate the degree to which PTK6 expression can be used as a prediction tool, time-dependent ROC curves were examined.

### 2.7. Analysis of Immune Microenvironment Based on Single-Sample Gene Set Enrichment Analysis (ssGSEA)

The purpose of ssGSEA (http://software.broadinstitute.org/gsea/msigdb/index.-jsp) is to categorize gene sets according to their shared biological function, chromosome mapping, and physiological regulation. We used ssGSEA to quantify the activity or enrichment level of immune cells, functions, or pathways in cancer samples in order to better understand the relationship between these factors.

### 2.8. Statistical Analysis

All statistical methods were accomplished by R (4.0.1). The differences between two groups were analyzed using the Student's *t*-test. Correlations between clinicopathological parameters and PTK6 expression were determined using chi square test. Survival curves were calculated using Kaplan-Meier estimates, and differences between groups were tested by log-rank test. A *p* < 0.05 was considered statistically significant.

## 3. Results

### 3.1. Identification of DEGs in BC

**Figure 1:**
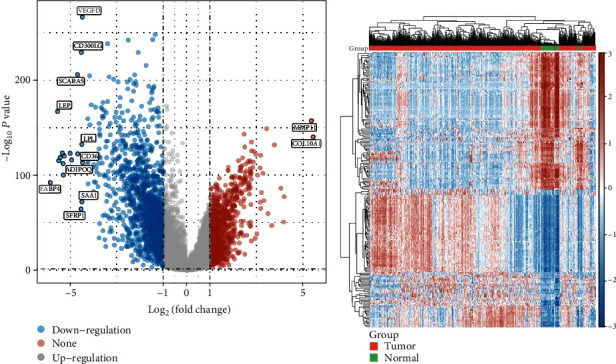
Differentially expressed genes between BC specimens and nontumor specimens, shown in (a) volcano map and (b) heat map.

In this work, a retrospective analysis of the data was performed on a total of 1109 breast cancer specimens and 113 nontumor specimens derived from the TCGA databases. The limma program was used to perform an analysis on the DEGs. A total of 2401 DEGs were obtained: 903 genes were significantly upregulated and 1498 genes were significantly downregulated (Figures 1(a) and 1(b)).

### 3.2. GO and KEGG Analyses

**Figure 2:**
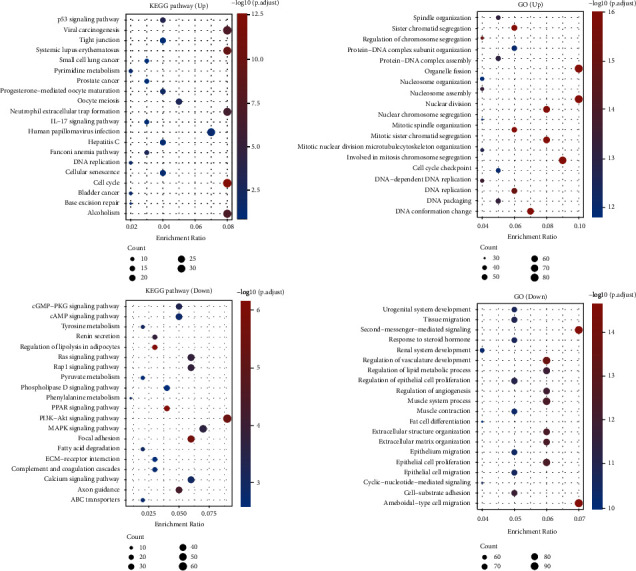
Functional enrichment analyses. (a) KEGG assays. (b) GO assays.

The functional roles of the DEGs between BC specimens and nontumor specimens from TGCA cohort were explored using GO enrichment and KEGG pathway analyses. KEGG analysis showed that the DEGs were mostly enriched in the p53 signaling pathway, viral carcinogenesis, systemic lupus erythematosus, IL-17 signaling pathway, cGMP-PKG signaling pathway, cAMP signaling pathway, and tyrosine metabolism (Figure 2(a)). GO analysis showed that the DEGs were mostly enriched in spindle organization, sister chromatid segregation, regulation of chromosome segregation, protein-DNA complex subunit organization, urogenital system development, tissue migration, second-messenger-mediated signaling, and response to steroid hormone (Figure 2(b)).

### 3.3. Differential Expression of PTK6 between Tumor and Normal Tissue Samples

**Figure 3:**
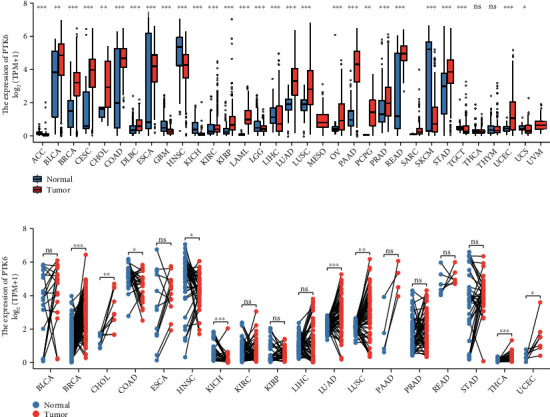
PTK6 profile across cancers. (a, b) The expression of PTK6 across normal and tumor tissues in the TCGA database via (a) unpaired *t*-test and (b) paired *t*-test. ^∗^*p* < 0.05, ^∗∗^*p* < 0.01, and ^∗∗∗^*p* < 0.001.

Among the DEGs, our attention focused on PTK6 whose function was rarely reported in BC. Firstly, we explored the expression of PTK6 in pan-cancer. As shown in Figures 3(a) and 3(b), we found that PTK6 exhibited a dysregulated level most types of tumors. Importantly, PTK6 expression was distinctly increased in most types of tumors. These findings indicated that PTK6 expression was elevated in a variety of cancers, suggesting that PTK6 may play a potentially crucial role in the detection of cancer.

### 3.4. The Expression of PTK6 in BC and Its Diagnostic Value

**Figure 4:**
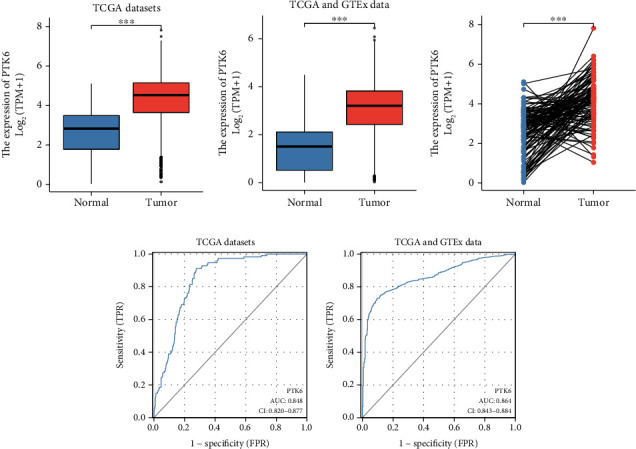
The expression and diagnostic value of PTK6 in BC patients. (a, b) PTK6 upregulation was confirmed in BC specimens compared with nontumor specimens in the TCGA datasets or the integrated GTEx database and TCGA database. (c) The results of unpaired *t*-test showing the overexpression of PTK6 in BC. (d, e) ROC curve of predictive value for BC specimens from the TCGA datasets or the integrated GTEx database and TCGA database. ^∗^*p* < 0.05, ^∗∗^*p* < 0.01, and ^∗∗∗^*p* < 0.001.

The expression of PTK6 in BC was the next thing that we looked at. When compared with nontumor specimens, the expression of PTK6 was noticeably upregulated in breast cancer samples (Figures 4(a) and 4(b)). The findings were also verified by the results of a paired *t*-test (Figure 4(c)). Subsequently, the diagnostic value of PTK6 for BC patients was explored. According to the ROC assays, high PTK6 expression had an AUC value of 0.848 (95% CI: 0.820 to 0.877) for BC in the TCGA datasets (Figure 4(d)). In addition, in the TCGA and GTEx datasets, high PTK6 expression had an AUC value of 0.864 (95% CI: 0.843 to 0.884) for BC (Figure 4(e)).

### 3.5. Overexpression of PTK6 Associated with Clinicopathologic Features of BC

**Figure 5:**
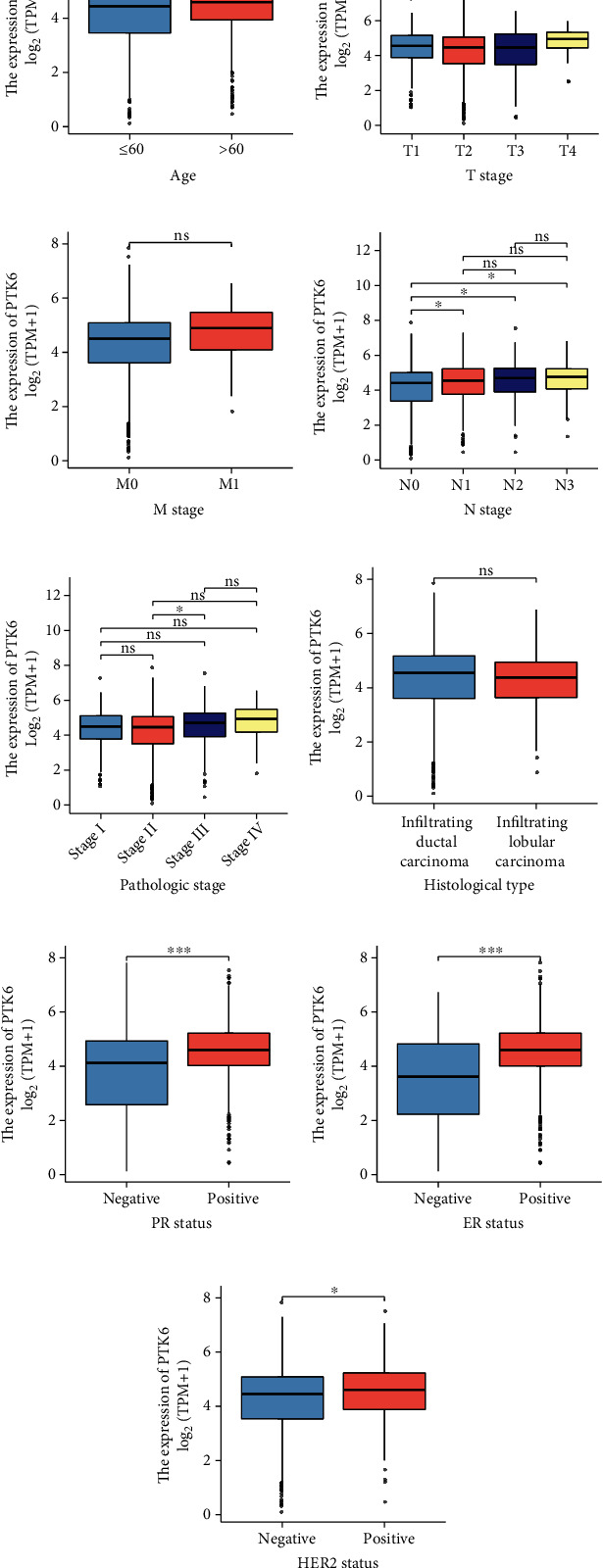
Differential PTK6 expression in various clinicopathological parameters, including (a) age, (b) T stage, (c) M stage, (d) N stage, (e) pathologic stage, (f) histological type, (g) PR status, (h) ER status, and (i) HER2 status. ^∗^*p* < 0.05, ^∗∗^*p* < 0.01, and ^∗∗∗^*p* < 0.001.

To explore the clinical significance of PTK6 expression in BC patients, we performed subgroup analysis. We observed that PTK6 exhibited a high level in BC patients with age > 60 than those with age < 60 (Figure 5(a)). Moreover, BC specimens with advanced TMN stage showed an increased expression of PTK6 (Figures 5(b)–5(e)). There was no significant difference in PTK6 expression between different histological types (Figure 5(f)). In addition, we analyzed the possible association between PTK6 expression and PR, ER, and HER2 status, finding that high PTK6 expression predicted a positive PR, ER, and HER2 status (Figures 5(g)–5(i)). Next, the relation between the expression level of PTK6 and the clinicopathological features was analyzed. The average expression levels of PTK6 were taken into account to divide BC patients into two groups, group with a high expression level and group with a low expression level. We observed that high expression of PTK6 associated with age, N stage, and pathologic stage (Table 1).

### 3.6. The Prognostic Value of PTK6 Expression in BC Patients

**Figure 6:**
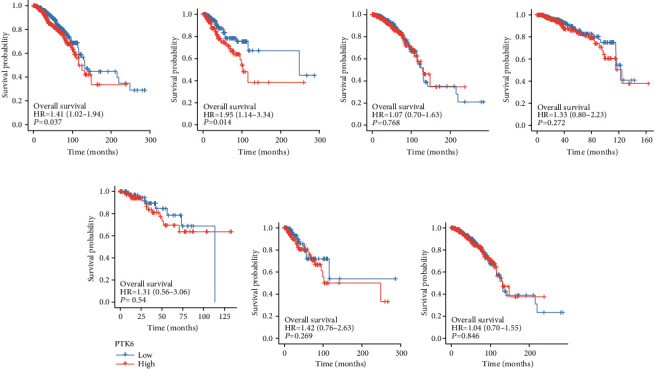
Survival curves were calculated using Kaplan-Meier estimates for the exploration of the possible influence of PTK6 expression on overall survival of BC patients. (a) All BC patients. (b) Patients with negative PR status. (c) Patients with positive PR status. (d) Patients with negative HER2 status. (e) Patients with positive HER2 status. (f) Patients with negative ER status. (g) Patients with positive ER status.

**Figure 7:**
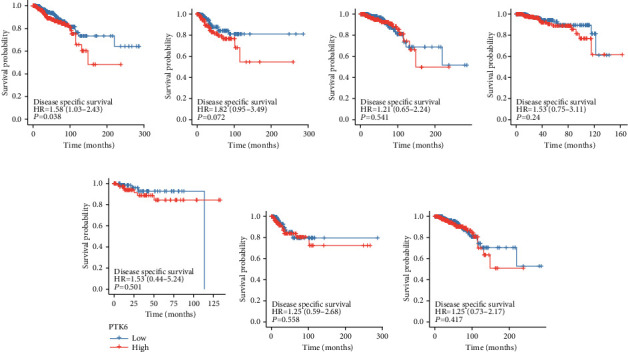
Survival curves were calculated using Kaplan-Meier assays for the exploration of the possible influence of PTK6 expression on disease-specific survival of BC patients. (a) All BC patients. (b) Patients with negative PR status. (c) Patients with positive PR status. (d) Patients with negative HER2 status. (e) Patients with positive HER2 status. (f) Patients with negative ER status. (g) Patients with positive ER status.

**Figure 8:**
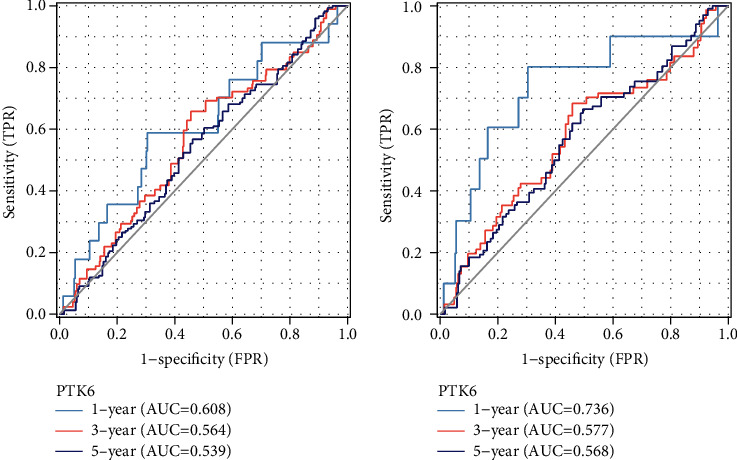
Time-dependent ROC curves of (a) overall survival and (b) disease-specific survival at 1, 3, and 5 years.

To further analyze the significance of PTK6 in terms of clinical prognosis, we performed the log-rank test. As shown in Figure 6(a), we found that the overall survival of BC patients with high PTK6 expression was significantly shorter than those with low PTK6 expression. Then, we performed subgroup analysis and found that high PTK6 expression was associated with shorter overall survival in BC patients with negative PR status (Figure 6(b)). However, we did not observe a possible association between PTK6 expression and BC patients with positive PR status, HER2 status, and ER status (Figures 6(c)–6(g)). In addition, we also confirmed that patients with high PTK6 expression showed a shorter disease-specific survival than those with low PTK6 expression (Figure 7(a)). However, the subgroup analysis showed PTK6 expression was associated with the prognosis of BC patients with different clinical status (Figures 7(b)–7(g)). To assess the accuracy of PTK6 expression predictions, we introduced a time-dependent ROC curve analysis. TCGA cohort showed a relatively high accuracy (Figures 8(a) and 8(b)).

### 3.7. Correlation between PTK6 Expression and Immune Infiltration

**Figure 9:**
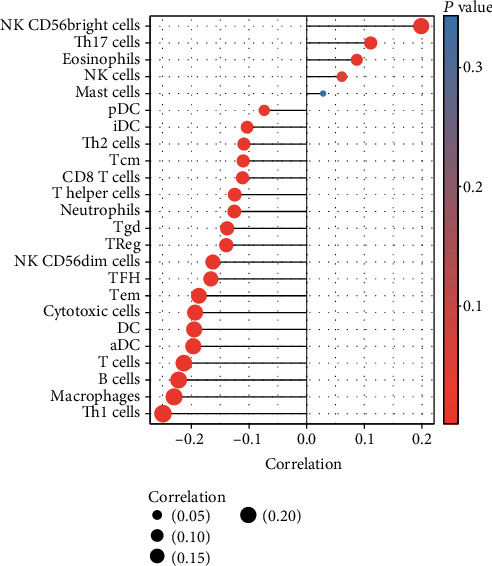
There was a correlation between the degree of PTK6 expression and immune infiltration in the BC microenvironment. The lollipop chart illustrates the correlation between the marker gene found in 24 immune cells and the expression level of the PTK6 gene.

**Figure 10:**
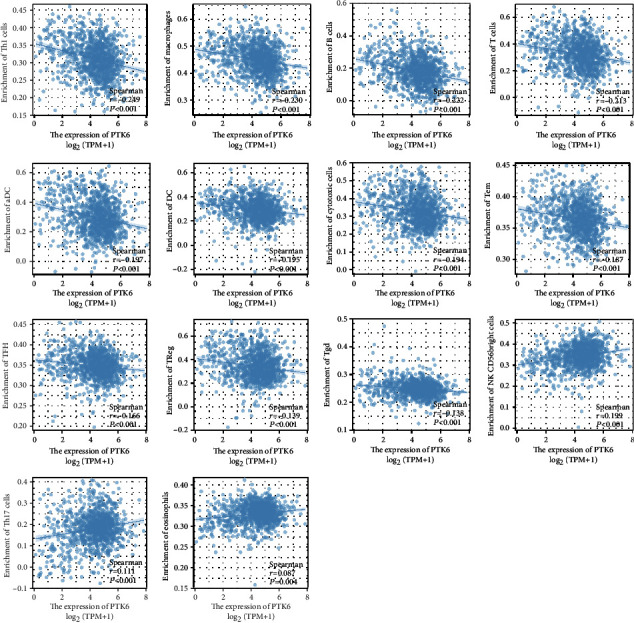
The correlations between the expression of PTK6 and immune cell. PTK6 expression was positively correlated with several immune cells.

A lollipop chart was used to illustrate the relationship between the levels of PTK6 and the number of immune cells (Figures 9 and 10). We found that PTK6 expression was positively correlated with the abundance of NK CD56bright cells and Th17 cells and negatively correlated with that of Th1 cells, macrophages, B cells, T cells, aDC, DC, cytotoxic cells, Tem, TFH, NK CD56dim cells, Treg, and Tgd. Our finding suggested PTK6 may be involved in the suppression of immune function in BC.

## 4. Discussion

Due to the fact that the population as a whole is getting older, we may anticipate that in the not-too-distant future, the percentage of senior people diagnosed with BC will most likely increase dramatically [29, 30]. However, because of a lack of appropriate data from clinical trials, there are still no clear therapy guidelines for older BC patients. This frequently results in patients receiving either an inadequate treatment or an excessive intervention, which in turn contributes to a bad prognosis. The research that is currently available suggests that senior patients with BC were treated less aggressively than their younger counterparts due to the fact that they had comorbidities and their performance status was falling. As a result, it is of utmost importance to assess the prognosis risk of senior patients diagnosed with BC in order to direct the therapeutic treatment decision and increase patients' overall survival.

To identify the BC-related genes, we firstly analyzed the TCGA datasets and identified 2401 DEGs. GO and KEGG assays suggested that the 2401 DEGs were involved in several tumor-related pathways. Among them, our attention focused on PTK6. PTK6 has been proven to have significant cancer-promoting effects in a number of different cancer types. For instance, it was observed that the expression levels of PTK6 and GAB1 were much higher in cervical cancer cell lines compared to those noted in normal cervical epithelial cells. Overexpression of GAB1 was able to offset the inhibitory effects of PTK6 knockdown on cervical cancer cells. Cervical cancer cells were protected against the progression of malignant cervical cancer when PTK6 expression was knocked down [31]. Cagle and his group showed that the overexpression of miR-214 in prostate cancer cells led to the induction of apoptosis, which in turn inhibited cell proliferation and the ability to form colonies. Expression of the microRNA miR-214 in prostate cancer cells was found to impede cell migration and invasion. The inhibition of cell growth caused by miR-214 was lessened when PTK6 expression was brought back to normal levels. In addition, the simultaneous inhibition of PTK6 by ibrutinib and miR-214 resulted in a considerable reduction in cell proliferation as well as cell survival [32]. PTK6 expression was found to be at a lower level in laryngeal squamous cell carcinoma tissues than in the neighboring noncancerous epithelial laryngeal tissues, and survival experiments demonstrated that PTK6 expression was a possible predictive factor for survival in laryngeal squamous cell carcinoma patients. Patients with laryngeal squamous cell carcinoma who had high expression levels of PTK6 had an overall and disease-free survival rate that was more favorable [33]. On the other hand, the roles of PTK6 in BC remained unknown for the most part. PTK6 expression was shown to be significantly elevated in the majority of tumor types, including BC, according to the findings of this investigation. The findings suggested that PTK6 might have a role in the promotion of tumors. In addition, ROC assays demonstrated that PTK6 was capable of screening BC specimens to differentiate them from nontumor specimens, underlining the diagnostic significance of PTK6 for BC patients. Patients who had high levels of PTK6 expression had a shorter overall survival and disease-specific survival than those who had low levels of PTK6 expression, according to the results of survival assays. This was the most important finding. Based on these findings, we hypothesized that PTK6 could serve as a diagnostic and prognostic biomarker for patients with BC.

Immunotherapy has reached an impressive level of effectiveness in the treatment of malignant solid tumors, providing an unmatched level of resolution in the fight against cancer [34, 35]. The efficacy of immunotherapy is sensitive to variations in time. It is helpful to understand the differences in the efficacy of immunotherapy and identify patients who will benefit the most from immunotherapy by investigating the characteristics of TME [36, 37]. There have been reports that immunotherapy, namely, ICB, can be beneficial to people with advanced forms of BC. The primary focus of cancer treatment has switched from the tumor itself to the immune system of the host as a direct result of the identification of immunological checkpoints and the positive therapeutic effects brought about by the use of immunosuppressants [38, 39]. When it comes to the successful implementation of ICB therapy, the antitumor immunity of the host and an inflammatory TME play critical roles in bringing about the desired outcome. The recent and major discovery of adaptive mechanisms of tumor resistance in TME, which may inhibit the implementation of tumor immunity, has been hailed as a significant milestone in the field of tumor immunotherapy [40, 41]. Immune cells that are present in the tumor microenvironment play an important function in tumor tissues, and there is growing evidence to support the clinicopathological significance of these cells in determining cancer patients' chances of survival and the effectiveness of their treatments. In this study, we found that PTK6 expression was positively correlated with the abundance of NK CD56bright cells and Th17 cells and negatively correlated with that of Th1 cells, macrophages, B cells, T cells, aDC, DC, cytotoxic cells, Tem, TFH, NK CD56dim cells, Treg, and Tgd. Our finding suggested PTK6 may be involved in the suppression of immune function in BC.

There were surely some limitations in the study. Firstly, to our knowledge, the clinical information contained in both the TCGA databases was limited. Secondly, the effectiveness of PTK6 still needs to be verified in a large number of external samples. Thirdly, laboratory-based experiments have not been conducted to validate whether the overexpression or knockdown of PTK6 suppressed the proliferation, migration, and invasion of BC cells.

## 5. Conclusion

We found that PTK6 was highly expressed in BC and predicted a poor prognosis. Identifying PTK6 as a biomarker for BC might help predict the prognosis and diagnosis of BC patients and could have important implications for immunotherapy.

## Figures and Tables

**Table 1:** The association between PTK6 expression and characteristics of patients suffering from BC.

Characteristic	Low expression of PTK6	High expression of PTK6	*p*
*n*	541	542	
Age, *n* (%)			0.035
≤60	318 (29.4%)	283 (26.1%)	
>60	223 (20.6%)	259 (23.9%)	
T stage, *n* (%)			0.051
T1	133 (12.3%)	144 (13.3%)	
T2	326 (30.2%)	303 (28.1%)	
T3	71 (6.6%)	68 (6.3%)	
T4	10 (0.9%)	25 (2.3%)	
N stage, *n* (%)			0.015
N0	283 (26.6%)	231 (21.7%)	
N1	172 (16.2%)	186 (17.5%)	
N2	48 (4.5%)	68 (6.4%)	
N3	33 (3.1%)	43 (4%)	
M stage, *n* (%)			0.254
M0	455 (49.3%)	447 (48.5%)	
M1	7 (0.8%)	13 (1.4%)	
Pathologic stage, *n* (%)			0.043
Stage I	93 (8.8%)	88 (8.3%)	
Stage II	326 (30.8%)	293 (27.6%)	
Stage III	105 (9.9%)	137 (12.9%)	
Stage IV	6 (0.6%)	12 (1.1%)	
Histological type, *n* (%)			0.152
Infiltrating ductal carcinoma	376 (38.5%)	396 (40.5%)	
Infiltrating lobular carcinoma	112 (11.5%)	93 (9.5%)	
Age, median (IQR)	56 (47, 66)	59 (50, 69)	0.001

## Data Availability

The datasets used and/or analyzed during the current study are available from the corresponding author on reasonable request.
